# Dynamic assembly of chromatin complexes during cellular senescence: implications for the growth arrest of human melanocytic nevi

**DOI:** 10.1111/j.1474-9726.2007.00308.x

**Published:** 2007-08

**Authors:** Debdutta Bandyopadhyay, Jonathan L Curry, Qiushi Lin, Hunter W Richards, Dahu Chen, Peter J Hornsby, Nikolai A Timchenko, Estela E Medrano

**Affiliations:** 1 Department of Molecular and Cellular Biology, Baylor College of Medicine Houston, TX 77030, USA; 2 Department of Dermatology, Baylor College of Medicine Houston, TX 77030, USA; 3 Department of Pathology, Baylor College of Medicine Houston, TX 77030, USA; 4 Department of Huffington Center on Aging, Baylor College of Medicine Houston, TX 77030, USA; 5 Department of Physiology and Sam and Ann Barshop Institute for Longevity and Aging Studies, University of Texas Health Science Center San Antonio, TX 78245, USA

**Keywords:** Brm1, HDAC1, human melanocytes, melanocytic nevi, melanoma, RB

## Abstract

The retinoblastoma (RB)/p16^INK4a^ pathway regulates senescence of human melanocytes in culture and oncogene-induced senescence of melanocytic nevi *in vivo*. This senescence response is likely due to chromatin modifications because RB complexes from senescent melanocytes contain increased levels of histone deacetylase (HDAC) activity and tethered HDAC1. Here we show that HDAC1 is prominently detected in p16^INK4a^-positive, senescent intradermal melanocytic nevi but not in proliferating, recurrent nevus cells that localize to the epidermal/dermal junction. To assess the role of HDAC1 in the senescence of melanocytes and nevi, we used tetracycline-based inducible expression systems in cultured melanocytic cells. We found that HDAC1 drives a sequential and cooperative activity of chromatin remodeling effectors, including transient recruitment of Brahma (Brm1) into RB/HDAC1 mega-complexes, formation of heterochromatin protein 1β (HP1β)/SUV39H1 foci, methylation of H3-K9, stable association of RB with chromatin and significant global heterochromatinization. These chromatin changes coincide with expression of typical markers of senescence, including the senescent-associated β-galactosidase marker. Notably, formation of RB/HP1β foci and early tethering of RB to chromatin depends on intact Brm1 ATPase activity. As cells reached senescence, ejection of Brm1 from chromatin coincided with its dissociation from HP1β/RB and relocalization to protein complexes of lower molecular weight. These results provide new insights into the role of the RB pathway in regulating cellular senescence and implicate HDAC1 as a likely mediator of early chromatin remodeling events.

## Introduction

Senescence has been implicated *in vivo* as a tumor-suppression mechanism in many cell types (Braig *et al*., 2005; Chen *et al*., 2005; Collado *et al*., 2005; Michaloglou *et al*., 2005). Melanocytes, the cell type from which melanoma arises, are specialized cells that synthesize and transfer the pigment melanin to surrounding keratinocytes, leading to skin pigmentation and protection against solar exposure. Progressive melanocyte dysfunction with aging may be associated with the dramatic increase in melanoma and nonmelanoma skin cancers in older individuals (Desai *et al*., 2006). Activating mutations of B-RAF trigger melanocyte proliferation and formation of melanocytic nevi (moles) (Pollock *et al*., 2003). However, further proliferation and risk of acquiring additional mutations is curtailed by the activation of the retinoblastoma (RB)/p16^INK4a^ but not the p53/p21 or p14^ARF^ pathways (Michaloglou *et al*., 2005) resulting in irreversible growth arrest of normal melanocytic nevi and senescence.

There are at least two different senescence mechanisms in human cells, a telomere-dependent pathway that is regulated by ATM/p53/p21 and a telomere-independent one often regulated by p16^INK4a^/RB (also referred to stasis). The RB family of proteins can induce repressive chromatin states around euchromatic promoters and have a direct role in the assembly of pericentric and telomeric heterochromatin domains (Gonzalo & Blasco, 2005). For example, Rb maintains histone H4 lysine 20 trimethylation (triMeH4K20) at constitutive heterochromatin domains (Gonzalo *et al*., 2005). Recent experimental evidence suggests that RB is a critical regulator of epigenetic changes during cellular senescence of melanocytes and fibroblasts (Bandyopadhyay *et al*., 2002; Narita *et al*., 2003). These data support an hypothesis proposed more than a quarter of century ago stating that aging of proliferating cells may result from genome reorganization occurring during the division cycle (Macieira-Coelho, 1980). RB represses E2F transcription factors by recruiting chromatin remodeling proteins including histone deacetylases (HDAC) and SWI/SNF complexes (Ferreira *et al*., 2001). Genetic studies in flies revealed that the HDACs Rpd3, an HDAC1 homolog (Johnson & Turner, 1999), and Sir2 have opposing effects in organismal lifespan; haploinsufficiency of Rpd3 extends Drosophila longevity by increasing the levels of Sir2 (Rogina *et al*., 2002) whereas greatly reduced levels of Rpd3 result in lethality (Mannervik & Levine, 1999).

Additional data supporting the role of chromatin remodeling in senescence of melanocytes include evidence that changes in p300 HDAC activity by overexpression of a dominant negative p300 protein or treatment with Lys-CoA (a specific chemical inhibitor of p300 HAT) result in early senescence of normal melanocytes, and surprisingly, also of melanoma cells (Bandyopadhyay *et al*., 2002). These studies suggested a model by which imbalances in the steady state ratio of HAT/HDAC induce extensive chromatin remodeling resulting in growth inhibition and cellular senescence (Bandyopadhyay *et al*., 2002).

Assembly of protein complexes in chromatin requires the use of adenosine triphosphate (ATP) as a source of energy to remodel nucleosomes (Johnson *et al*., 2005). Such energy can be provided by a variety of chromatin remodeling complexes including SWI/SNF, nucleosome remodeling factor (NURF), chromatin accessibility complex (CHRAC), and ATP-dependent chromatin-assembly factor (ACF). Brahma-related gene 1 (Brg1) and Brahma (Brm1) are two members of SWI/SNF complexes that contain a highly conserved ATPase domain and a C-terminal bromodomain. Evidence implicating members of SWI/SNF chromatin remodeling complexes in aging is limited but suggestive. For example, Brg1/RB complexes can induce cell cycle arrest (Dunaief *et al*., 1994) and a senescent-like morphology in the human adrenal cortex carcinoma-derived cell line SW13 (Shanahan *et al*., 1999). More significantly, repressive complexes containing Brm1, RB, and C/EBPα regulate liver aging in mice (Iakova *et al*., 2003). In this work, we present evidence that increased HDAC1 levels and activity initiate a sequential and cooperative assembly of chromatin effector proteins leading to the formation of early, transient Brm1/RB/Hp1β complexes, stable association of RB with chromatin, formation of RB/HP1β and HP1β/SUV39h1 foci, progressive heterochromatinization, and ultimately irreversible cell cycle arrest and senescence.

## Results

### HDAC1 drives senescence in melanocytic cells

Human intradermal melanocytic nevi are essentially senescent entities resulting from the oncogenic activation and limited proliferation of melanocytes by mutated, constitutively active BRAF^E600^ kinase (Michaloglou *et al*., 2005). In contrast, recurrent melanocytic nevi are proliferations of cytologically atypical melanocytes localized to the epidermis and organized as nests and as single cells with areas of upward epidermal migration. These lesions may contain residual senescent melanocytic nevi adjacent to the area of recurrence. Thus, recurrent melanocytic nevi provide a unique *in vivo* system to study proliferating melanocytes that have escaped cellular senescence and have re-entered the cell cycle.

**Fig. 1:**
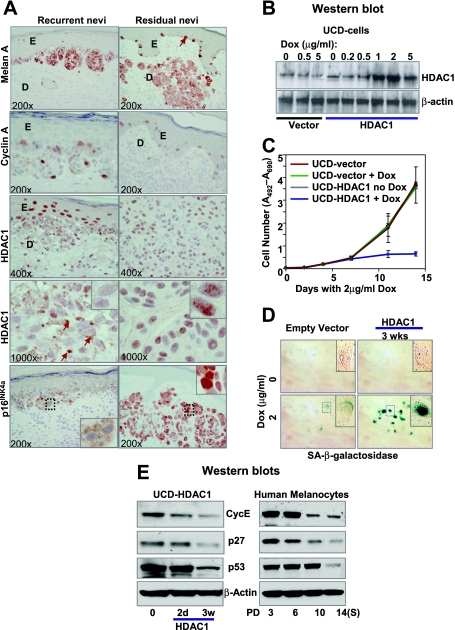
Overexpression of HDAC1 results in expression of senescent makers *in vitro* and correlates with senescence of intradermal melanocytic nevi *in vivo*. (A) HDAC1 is prominently detected in residual, senescent nevi but not in proliferating recurrent nevi. Melan-A highlights recurrent melanocytes in the epidermis and residual dermal nests of melanocytes (left and right column, first row). Note the positive staining of normal intra-epidermal melanocytes with Melan-A along the dermal–epidermal (right column, first row, red arrow). Recurrent melanocytic nevus show confluent proliferation of atypical melanocytes in the epidermis along the dermal–epidermal junction (E) and underlying scar tissue in the dermis (D, left column). Corresponding residual dermal nests of nevomelanocytes are located adjacent to the scar tissue and separated from the area of recurrence (D, right column). Recurrent nevi and scattered basal keratinocytes exhibit cyclin A immunoreactivity whereas the residual nevi are virtually cyclin A negative. There is absence of HDAC1 staining in the recurrent nevus cells along the dermal–epidermal junction (a white line was drawn to highlight a nevus nest) compared to the nuclear expression of HDAC1 in epidermal keratinocytes, which serve as a positive internal control (left column, third row). A higher magnification of recurrent melanocytes confirms that the nuclei of the recurrent nevi are essentially HDAC1 negative. Brown arrows highlight melanin pigment (left column, fourth row). In contrast, residual dermal nests of nevomelanocytes display positive nuclear immunoreactivity for HDAC1 in foci-like structures (right column, third and fourth row and insert). p16^INK4a^ staining demonstrated focal weak immunoreactivity in the recurrent nevi (left column, last row and insert) compared to strong nuclear and cytoplasmic expression staining in the residual nevus cells (right column, last row and insert). Virtually identical results were obtained in two additional sets of recurrent/residual nevi and in three independent intradermal melanocytic nevi. (B) Western blot showing inducible HDAC1 levels by the Tet-On system in UCD-Mel-N melanoma cell line. Numbers above the figure indicate Dox concentrations. (C) Growth curves of UCD-Empty vector control and UCD-HDAC1 cells before and after treatment with Dox. (D) Induction of the SA-β-gal marker in irreversibly quiescent cells 3 week after HDAC1 induction. Software enhancement of color was used to show the cell morphology of the SA-β-gal-negative cells in the inserts of the right and left upper figures. (E) Senescence induced by HDAC1 reproduces features of normal melanocyte senescence. Protein extracts from cells were electrophoresed, transferred to nitrocellulose and probed with the antibodies shown in the figure. PD, population doublings; S, senescent melanocytes (normal melanocytes usually senesce after 12–14 PD).

High levels and activity of HDAC1 associate with RB complexes in senescent melanocytes and are responsible for silencing the *cyclin E* gene in these cells (Bandyopadhyay *et al*., 2002). To determine whether HDAC1 expression is associated with senescence of melanocytic nevi *in vivo* and normal melanocytes in culture, we used immunohistochemistry in human specimens of recurrent melanocytic nevi. We show that cyclin A positive, proliferating melanocytes localized along the dermal–epidermal junction did not display detectable HDAC1 immunoreactivity (Fig. 1A and third and fourth rows ‘recurrent nevi’). Differentiating, HDAC1-positive keratinocytes in the epidermal (E) layer served as an internal control for antibody immunoreactivity. In contrast, the residual, cyclin A negative, dermal nests of melanocytes (D) demonstrated prominent nuclear HDAC1 staining in foci-like structures. These results are consistent with previous data showing that HDAC1 is a potent cyclin A repressor (Stiegler *et al*., 1998). Staining with an anti-p16^INK4a^ antibody demonstrated scattered, weak immunoreactivity in recurrent nevus cells whereas a strong, diffuse nuclear and cytoplasmic immunoreactivity (Gray-Schopfer *et al*., 2006; Nilsson & Landberg, 2006) was observed in the residual dermal nests of melanocytes (Fig. 1A, last row). In agreement with previous data (Michaloglou *et al*., 2005), we were unable to detect p21^Waf1^ in the senescent nevi (data not shown).

To investigate whether HDAC1 affects the onset and/or maintenance of senescence, we used a tetracycline regulated HDAC1-inducible system. Engineering adequate HDAC1 regulation and studying its consequences is not feasible in normal human melanocyte cultures that are capable of only 12–14 population doublings before the onset of senescence (Bennett & Medrano, 2002). Therefore, we used the melanoma cell line UCD-Mel-J, as it expresses wild-type p53 and RB, but does not express p16^INK4a^ (E. E. Medrano, unpublished results). The Tet-regulated UCD-derivative (UCD-HDAC1) cell line expressed HDAC1 when treated with doxycycline (Dox), a potent tetracycline analog (Fig. 1B). HDAC1 induction was not observed after Dox treatment of cells transfected with an empty vector. Total HDAC activity also increased after addition of 2 µg mL^−1^ Dox (Supplementary Fig. S1-A); thus, the ectopically expressed HDAC1 was enzymatically proficient. An MTT-cell proliferation assay demonstrated that 10–14 days after HDAC1 induction, UCD-HDAC1 cells became growth arrested (Fig. 1C, blue line), whereas uninduced UCD-HDAC1 cells, or UCD cells harboring an empty vector in the presence or absence of Dox, displayed normal exponential growth (Fig. 1C, gray, red, and green lines, respectively). A bromodeoxyuridine incorporation assay confirmed that induction of HDAC1 paralleled a concomitant decrease in the number of cells in the S phase of the cell cycle (Supplementary Fig. S1-B).

After 3 weeks of sustained HDAC1 levels the cells became irreversibly arrested (Fig. 1D). These cells did not resume growth upon Dox removal (Supplementary Fig. S1-C), and continued to display typical markers of normal melanocyte senescence (Bennett & Medrano, 2002) including expression of the senescent-associated β-galactosidase (SA-β-gal) marker (Fig. 1D), a flat morphology, and aberrant cytokinesis (presence of multinucleated cells; data not shown).

Paradoxically, HDAC1 can negatively regulate transcription of a number of cell cycle genes associated with proliferation as well as tumor suppression. Chromatin immunoprecipitation (ChIP) assays previously demonstrated that silencing of the *cyclin E* gene is associated with binding of HDAC1 complexes to its promoter in senescent melanocytes (Bandyopadhyay *et al*., 2002). HDAC1 is also a negative regulator of p27^KIP-1^ (Lagger *et al*., 2002) and p53 (Ito *et al*., 2002; Lagger *et al*., 2003) transcription. HDAC1-induced senescence (Fig. 1E) faithfully reproduced the down-regulation of cyclin E, p53, and p27^KIP-1^ previously observed during normal melanocyte senescence (Bandyopadhyay *et al*., 2001; Bennett & Medrano, 2002). Senescent melanocytic nevi do not display telomere attrition and do not show evidence for up-regulation of p53 or p21^Waf-1^ (Michaloglou *et al*., 2005). Similarly, HDAC1 did not elicit appreciable changes in telomerase activity (Supplementary Fig. S1-E). This suggests that loss of telomerase-mediated telomere elongation was not a proliferation-limiting event in UCD-HDAC1 cells.

### HDAC1-induced signatures of progressive heterochromatinization: formation of Rb/HP1β and HP1β/SUV39H1 nuclear foci, trimethylation of H3-K9, and stable association of Rb with chromatin

**Fig. 2:**
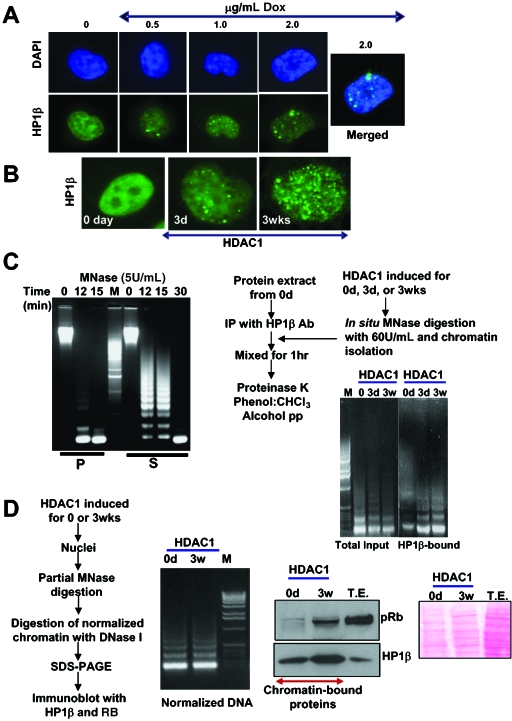
Overexpression of HDAC1 triggers a time-dependent increase in HP1β foci and association of RB and HP1β with chromatin. (A) HDAC1-induced cells show formation of HP1β foci in a dose-dependent manner. The cells were processed for immunocytochemistry 3 days after Dox treatments. (B) HDAC1 induces HP1β foci in a time-dependent manner. The experiments shown herein and in subsequent figures used 2 µg mL^−1^ Dox to induce HDAC1. (C) Increased affinity of HP1β with senescent chromatin. Left panel: chromatin from senescent melanocytes shows increased resistance to micrococcal nuclease (MNase) digestion (see Experimental procedures). Right panel: an *in vitro* assay (Experimental procedures) using MNase-digested chromatin with a size range of 146 bp–2 kbp demonstrated that HP1β has increased affinity for senescent chromatin. (D) A cell-based assay (Experimental procedures) confirmed and extended results from the *in vitro* assay by showing that HDAC1 increases the association of both HP1β and RB with chromatin. M, DNA marker. T.E., total protein extract from uninduced UCD-HDAC1 cells. Right panel: Ponceau red staining indicating protein loading.

Senescent fibroblasts display heterochromatic structures named senescence-associated heterochromatic foci (SAHF) (Narita *et al*., 2003), which contain the histone variant macro H2A (Zhang *et al*., 2005). Formation of such structures coincides with the recruitment of RB into protein complexes containing the heterochromatin 1 binding protein 1γ (HP1γ) (Narita *et al*., 2003; Zhang *et al*., 2007). We found that senescent normal human melanocytes displayed prominent HP1β foci compared to the nucleoplasm-dispersed HP1β in proliferating cells (Supplementary Fig. S2). Overexpression of HDAC1 induced nuclear HP1β foci in a dose- and time-dependent manner (Fig. 2A,B). Formation of HP1β foci was HDAC1 dependent, as no foci were observed after treating vector-only cells with Dox (data not shown). As the transition from euchromatin to heterochromatin is gradual and requires multiple cell divisions (Katan-Khaykovich & Struhl, 2005), early formation of a relatively low number of HP1β foci in the proliferative phase after HDAC1 induction (Fig. 2B – ‘3 days’) likely represents chromatin remodeling events and not SAHFs. Conversely, presence of a large number of HP1β foci (Fig. 2B– ‘3 weeks’) may be indicative of higher order chromatin structures present in the senescent cells (Taddei *et al*., 2005, 2006).

**Fig. 4:**
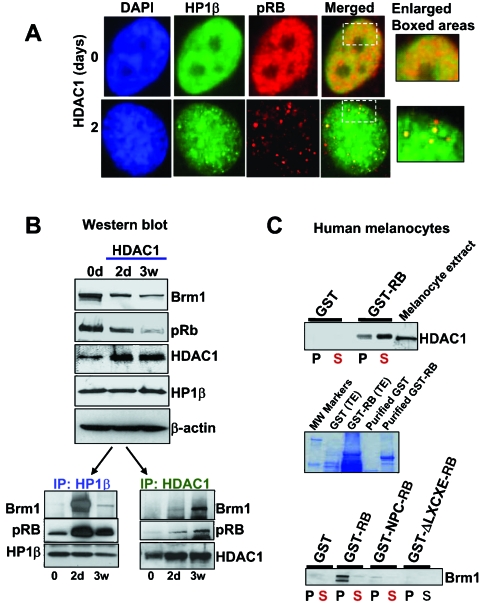
Induction of HDAC1 results in transient association of Brm1 with RB/HP1 complexes. (A) HDAC1 induces the association of HP1β with RB in nuclear foci. The boxes indicate areas zoomed to visualize the foci present in HDAC1-induced but not in control cells. The UCD-HDAC1 cells were treated as described in the legend of Fig. 3. (B) Western blot showing HDAC1 induction and concomitant changes in Brm1, RB, HDAC1, and HP1β protein levels. Immunoprecipitations with HP1β and HDAC1 antibodies suggest that large amounts of Brm1 are transiently associated with RB/HP1β complexes. (C) GST pull-down assays using extracts from proliferating (P) and senescent (S) melanocytes (see text for details). Upper panel: Increased association of HDAC1 with RB in senescent melanocytes. Middle panel: coomassie blue staining of total extracts (TE) and purified GST and GST-RB proteins. Bottom panel: GST-RB pulls down Brm1 from proliferating melanocytes whereas minimal levels are pulled down from senescent cultures. GST-NPC-RB and GST-LXCXE-RB were used as negative controls.

DNA from *ras*-induced senescent fibroblasts (Narita *et al*., 2003), or from normal senescent melanocytes, displays increased resistance to micrococcal nuclease digestion compared to proliferating cells (Fig. 2C, left panel). An *in vitro* assay (Fig. 2C, right panel) demonstrated that HP1β displayed increased association with chromatin. A cell-based assay indicated that both RB and HP1β became strongly tethered with chromatin in the senescent cells (Fig. 2D). Importantly, these results are independent of the respective protein levels; RB protein levels decrease whereas HP1β does not change in the senescent cells compared to the uninduced proliferating cells (Fig. 4B).

**Fig. 3:**
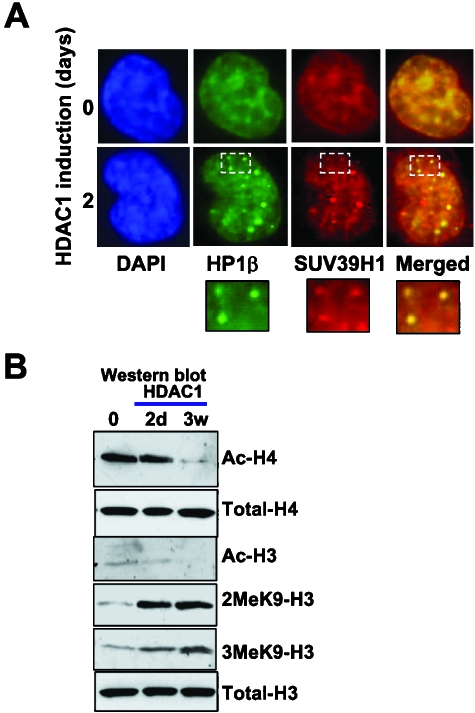
HDAC1 induces features of heterochromatinization. (A) Colocalization of HP1β and SUV39H1 in nuclear foci. UCD-HDAC1 cells were left untreated or treated with 2 µg mL^−1^ doxycline for 2 days, fixed and processed for immunofluorescence using HP1β and SUV39H1 as described in Experimental procedures. The boxes indicate areas zoomed (bottom figures) to show colocalization of HP1β with SUV39H1. (B) HDAC1 induces deacetylation of H4 and H3, and di- and trimethylation of H3-K9. 2MeK9-H3, dimethylated K9 on histone H3; 3MeK9-H3, trimethylated K9 on histone H3.

Rb interacts with HDAC1 through the ‘pocket’ domain resulting in modifications of the chromatin structure (Brehm *et al*., 1998). RB is necessary to direct methylation of histone H3-K9 by the histone methyltransferase SUV39H1 (Nielsen *et al*., 2001). In turn, recruitment of HP1 proteins to chromatin requires H3-K9 methylation and direct protein–protein association between SUV39H1 and HP1 (Stewart *et al*., 2005). Induction of HDAC1 resulted in the rapid redistribution and colocalization of SUV39H1 and HP1β in nuclear foci, whereas proliferating cells mostly displayed unstructured localization of both proteins (Fig. 3A). HDAC1 also favored H3-K9 di- and trimethylation (Fig. 3B). These events, which are consistent with features of heterochromatinization in skin fibroblasts from aging baboons (Herbig *et al*., 2006), are likely mediated by the direct association of HDAC1 with SUV39H1 (Vaute *et al*., 2002). Methylation of H3-K9 appears to be a two-phase dynamic event associated with HDAC1 induction (Fig. 3B). Whereas dimethylation followed rapid kinetics and correlated with formation of HP1β/SUV39H1 foci, trimethylation of H3-K9 occurred with slower kinetics and only reached a plateau in senescent cells. These data suggest that H3-K9 trimethylation may be regulated by another histone methyltransferase in the senescent cells, or alternatively, by a different set of proteins regulating SUV39H1 activity at the onset of senescence. Finally, extensive heterochromatinization after prolonged HDAC1 induction may explain the irreversibility of the senescent state upon Dox removal, as most histone acetyltransferases will not have access to the higher order chromatin regions.

### Transient association of Brm1 with Rb and HP1β precedes growth inhibition driven by HDAC1

The repressive functions of RB require its association with several proteins including HDAC1 (Brehm *et al*., 1998; Magnaghi-Jaulin *et al*., 1998), HP1 (Nielsen *et al*., 2001), and Brm1 (Trouche *et al*., 1997). To molecularly define the role of RB-associated proteins during senescence driven by HDAC1, we analyzed changes in expression and protein–protein associations of Brm1, RB, and HP1β before and after Dox treatments. HDAC1 initiated a rapid distribution of nucleoplasmic RB into nuclear foci, colocalizing with HP1β (Fig. 4A). Total levels of Brm1 and RB declined rapidly after HDAC1 induction, an event that was even more pronounced in the senescent cells (Fig. 4B). Notably, immunoprecipitation with an HP1β antibody showed a dramatic and transient association of Brm1 with Rb and HP1β (Fig. 4B, left panel), whereas immunoprecipitation with an HDAC1 antibody showed a time-dependent increase of both Brm1 and RB association with HDAC1 (Fig. 4B, right panel). Limited amounts of BRM1 associated with HDAC1 suggest that the bulk of this protein associated with complexes containing HP1β (Fig. B, left panel). Glutathione S-transferase (GST) pull-down assays showed that RB strongly interacts with HDAC1 in senescent melanocytes (Fig. 4C, upper panel), whereas it associates with Brm1 in proliferating cells but only minimally in senescent cells (Fig. 4C, bottom panel). A coomasie gel (Fig. 4C, middle panel) indicated GST-RB protein expression. We noticed that the Brm1 antibody detected a prominent doublet in proliferating melanocytes and UCD-HDAC1 cells. Such a doublet, which was also detected by a different Brm1 antibody in NIH3T3 cells (Harikrishnan *et al*., 2005), could indicate Brm1 post-translational modifications. Two negative controls demonstrated the specificity of the GST assays. A RB mutant lacking 14 of the 15 conserved cyclin-dependent kinase consensus phosphorylation sites (RB-NPC) (Chew *et al*., 1998) showed negligible association with Brm1 compared to wild-type RB. This suggests that as cells senesce, dephosphorylation of RB (Fig. 4B) impairs its binding to Brm1. A second RB mutant (RBΔLXCXE) confirmed that the LXCXE binding pocket is required for the association of RB with Brm1 (Trouche *et al*., 1997). In conclusion, the GST pull-down assays in normal senescent melanocytes support the findings using the HDAC1-inducible model in melanoma cells (Fig. 4B).

**Fig. 5:**
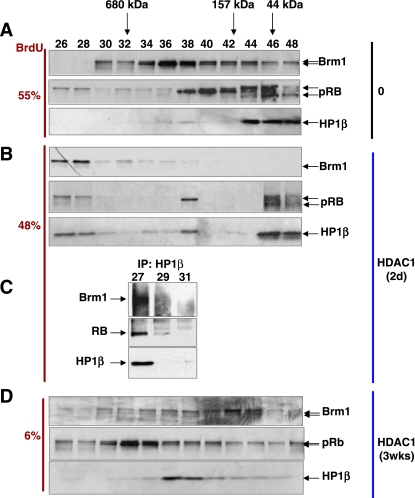
Formation of transient mega-complexes containing Brm1/RB/HP1β precedes growth inhibition. HPLC fractionation, Western blotting and coimmunoprecipitations of control cells (0 day) and cells induced to express HDAC1 for 2 days or 3 weeks (see Experimental procedures). IP, immunoprecipitation.

To define the kinetics of Brm1 complex formation, we used size exclusion chromatography and Western blotting with antibodies to Brm1, RB, and HP1β. In uninduced cells, HP1β migrated mostly as a monomer, whereas Brm1 was widely distributed indicating that this protein forms multiprotein complexes with diverse partners (Fig. 5A). Induction of HDAC1 elicited a rapid redistribution of Brm1, HP1β, and RB to high molecular weigh fractions (Fig. 5B). Immunoprecipitation with an HP1β antibody (Fig. 5C) confirmed the association of HP1β with RB and Brm1. This mega-complex likely contains additional proteins as the sum of the molecular weights of Brm1, HP1β, and RB (~310 kDa) is significantly lower than that of the observed complex (~1 × 10^6^ kDa). Significantly, this high molecular weight complex, which was formed when the cells were still proliferating (Fig. 1B), was undetectable in the senescent cells (Fig. 5D). At this time, the bulk of RB had moved to lower molecular weight fractions (~680 kDa), partially colocalizing with Brm1 and HP1β. Together, these experiments suggest that the recruitment of RB to protein complexes is a dynamic, time-dependent event mediated by the increased activity of HDAC1.

### Transient expression of a dominant negative Brm1 (dnBrm1) prevents the association of RB and HP1β with chromatin and formation of HP1β nuclear foci

**Fig. 6:**
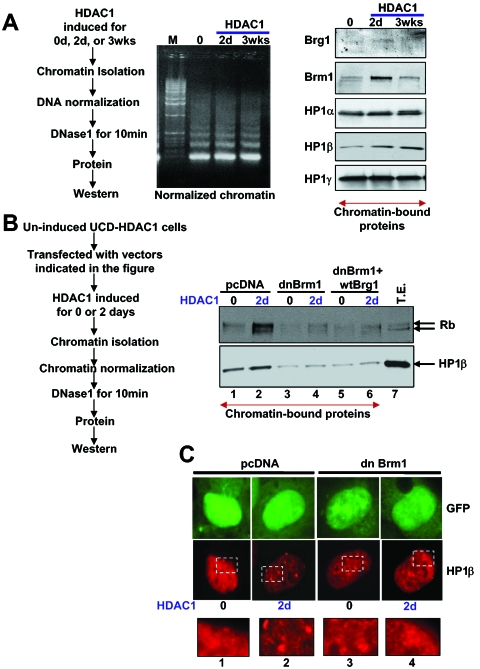
Brm1 ATPase activity is required for the association of RB and HP1β with chromatin and formation of HP1β foci. (A) Transient association of Brm1 but not Brg1 with chromatin 2 days after HDAC1 induction. (B) Transient expression of dnBrm1 prevented the association of RB and HP1β with chromatin. Transfection of wild-type Brg1, a highly homologous Brm1 protein, did not compensate dnBrm1 dominant negative activity. (C) dnBrm1 prevents formation of HP1β foci. UCD-HDAC1 cells were transiently transfected with pcDNA or dnBrm1. Cotransfection with a GFP-expressing plasmid was used to identify cells expressing an empty vector (pcDNA) or dnBrm1. After 8 h, cells were left untreated or treated with Dox for 2 days (to induce HDAC1). Coverslips were processed for immunofluorescence as described in Experimental procedures. The rectangular marquees indicate areas zoomed at the bottom to show that dnBrm1 curtails foci induced by HDAC1 (compare Fig. 6C second panel with Fig. 6C fourth panel).

To test whether transient formation of the Brm1/RB/HP1β complex coincides with the association of Brm1 with chromatin, cells were induced to express HDAC1 for 2 days and 3 weeks. Brm1 strongly, though transiently, associated with chromatin after HDAC1 induction (Fig. 6A, right panel). Yet, in contrast with the stable association of RB with chromatin (compare Fig. 2D with 6B), Brm1 was minimally detected in the chromatin of senescent cells. Interestingly, Brg1, a Brm1-related ATPase, showed minimal association with chromatin. Together, the results suggest that the chromatin-remodeling activity of Brm1 is an early event likely required for nucleosomal remodeling in preparation for trimethylation of H3-K9 and irreversible heterochromatinization. Thus, it is likely that Brm1 activity may be dispensable for the maintenance of repressive heterochromatin. Notably, a similar exclusion of Brm1 from condensed chromatin operates in mitotic cells (Muchardt *et al*., 1996). Stable recruitment of HP1 to chromatin requires two interactions: association of HP1 with methylated H3-K9 and direct protein–protein interaction between HP1 and SUV39H1 (Stewart *et al*., 2005). Increased HDAC1 activity leads to elevated HP1β recruitment to chromatin in a time-dependent manner that peaked in the senescent cells (Fig. 6A). It has been previously shown that the three HP1 proteins (α, β, and γ) localize to DNA foci in senescent fibroblasts (Narita *et al*., 2003). However, neither HP1α nor HP1γ showed appreciable changes in chromatin association after HDAC1 induction (Fig. 6A) in melanoma cells.

To test whether the ATP-dependent chromatin-remodeling activity of Brm1 is required for the transient association of RB and HP1β to chromatin, we transfected an empty vector control, a dominant negative, ATPase-dead Brm1 (dnBRM1) mutant that lacks chromatin-remodeling activity (Bourachot *et al*., 1999) or dnBrm1 plus Brg1, in UCD-HDAC1 cells before or after induction of HDAC1 for 2 days. Remarkably, the dnBRM1 mutant almost completely prevented the association of both RB and HP1β with chromatin (compare Fig. 6B, lane 4 with lane 2). Consistent with previous results showing that Brg1 and Brm complexes regulate distinct cellular processes through protein–protein interactions that are specific for each ATPase (Kadam & Emerson, 2003), cotransfection of dnBrm1 with Brg1 did not compensate dnBrm1 activity (compare Fig. 6C, lane 6 with lane 2). A functional Brm1 ATPase activity was also required for the redistribution of nucleoplasmic HP1β into nuclear foci [Fig. 6C, compare HDAC1 induction at 2 days without (second panel) and with (fourth panel) dnBrm1]. Finally, our results support and extend the notion that alterations in DNA-histone contacts are regulated by the ATPase activity of SWI/SNF complexes resulting in conformational changes within the nucleosome (Johnson *et al*., 2005).

## Discussion

The tumor suppressor Rb functions as a classical gatekeeper by inducing irreversible growth arrest and senescence of potentially cancerous cells (Kinzler & Vogelstein, 1997; Campisi, 2005; Johnson *et al*., 2005). We have identified HDAC1 as a critical mediator of chromatin remodeling events that take place prior to overt senescence. Of most importance, our data suggest that induction of HDAC1 may be a pivotal step in oncogene-induced senescence of melanocytic nevi (Fig. 1).

**Fig. 7:**
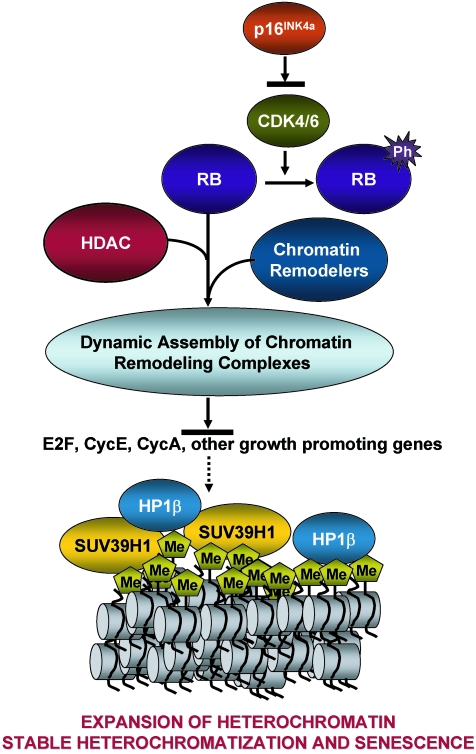
Changes in the stoichiometry of RB/HDAC1 complexes regulate the onset of senescence. We propose a model by which up-regulation of p16^INK4a^ converts RB into its active, nonphosphorylated form which in turns recruits HDAC1, initiating a chain of dynamic chromatin events eventually leading to expansion of heterochromatin and cellular senescence (see text). A dotted arrow indicates that insertion of histone variants, changes in the nucleosome structure and yet to be defined molecular events including post-translational modifications of chromatin regulators contribute to the formation of stable heterochromatization and irreversibility of the senescent state.

Based on experiments described in this article, we propose a model (Fig. 7) by which up-regulation of p16^INK4a^ triggers RB dephosphorylation and recruitment of increased levels of HDAC1. Changes in the stoichiometry and dynamics of RB macromolecular complexes, by up-regulation of HDAC1 via increased transcription and/or protein stabilization (Fig. 1), or by increased use of the cell's steady-state HDAC1 pool, in turn initiate a chain of events leading to chromatin remodeling and silencing of growth-promoting genes. At an early stage, reduction of HDAC1 levels (Supplementary Fig. S1) will antagonize RB-mediated repression and result in resumption of the cell cycle. Conversely, severe, chronic HDAC1 overexpression will lead to pervasive tethering of HP1β and RB with chromatin, heterochromatin expansion and cellular senescence. Finally, this model accounts for the results described herein, namely, overexpression of HDAC1 can overcome the loss of p16^INK4a^ and induce senescence in human melanoma cells. Studies in progress are aimed at determining the molecular mechanism(s) of HDAC1 up-regulation in melanocytic cells.

### HDAC1 regulates multiple events leading to heterochromatinization

Gene silencing acts in a regional rather than promoter- or sequence-specific manner to generate large DNA domains that are inaccessible to DNA binding factors (Moazed, 2001). RB repressive complexes transiently associate with chromatin during cell cycle arrest (Angus *et al*., 2004). However, RB is strongly and stably associated with chromatin in senescent cells (Fig. 2D). Changes in the stoichiometry of RB/HDAC1 complexes result in trimethylation of H3-K9, multiple HP1β foci and senescence (Figs 2–4). Such changes are expected to be structurally different from those observed in cells entering reversible quiescence (Barbie *et al*., 2004), or after short-term (2 days) HDAC1 induction, and associated with irreversible repression of E2F-regulated genes including cyclins E and A via formation of E2F4/HDAC1/RB complexes (Bandyopadhyay *et al*., 2002; Narita *et al*., 2003).

HDAC1 also induced a rapid relocalization of HP1β to DNA foci and dimethylation of H3-K9 (Fig. 3), an event associated with hypoacetylation in the coding regions of human genes (Miao & Natarajan, 2005). As dimethylated H3-K9 is diffusely scattered throughout silent euchromatin (Rice *et al*., 2003), our data suggests that increased HDAC1 activity may expand such compartments by switching active genes into silent euchromatin.

HP1 is transiently recruited to promyelocytic leukemia bodies prior to its association with SAHF in human fibroblasts (Zhang *et al*., 2005). The discrete number of HP1β foci observed 2 days after HDAC1 induction suggest that these foci colocalize with promyelocytic leukemia bodies (Hayakawa *et al*., 2003) as the cells were still in the proliferative phase that preceded senescence (Figs 1 and 2). Alternatively, early formation of HP1β foci may be indicative of active chromatin reorganization in a manner similar to the one observed during the final rounds or replication that precede cell cycle exit triggered by nutrient deprivation or after contact inhibition (Barbie *et al*., 2004). Such cell cycle exit coincides with the reversible phase of cell cycle arrest induced by HDAC1 (Supplementary Fig. 1C) and RB (Angus *et al*., 2004).

Mammalian HP1β forms stable complexes with SUV39H1 (Aagaard *et al*., 1999). In fact, SUV39H1 but not G9a, also an H3-K9 methyltransferase, mediates the recruitment of HP1 to chromatin (Stewart *et al*., 2005). In turn, the SET domain of SUV39H1 appears to stabilize the association of SUV39H1 with heterochromatin and also acts as a platform for the interaction with other heterochromatin proteins. The reciprocal changes in the localization of SUV39H1 and HP1β with chromatin (Fig. 3A) suggest that HDAC1 facilitates the recruitment of these proteins to chromatin (Krouwels *et al*., 2005). Future studies will address whether the DNA methyltransferases including Dnmt1 and Dnmt3a, which are known to associate with HP1 and SUV39H1 (Fuks *et al*., 2003), are also required for the reinforcement of repressive chromatin in senescent cells.

### Brm1 drives early localization of RB and HP1β with chromatin and formation of HP1β foci

SWI/SNF factors appear to influence higher-order chromatin structures by several means including transferring the entire core histone octamer to another region of DNA, disrupting nucleosomes, changing nucleosome positioning, switching between relaxed to condensed chromatin, bridging nucleosomes with 30 nm fibers, and/or attaching chromatin fibers to nuclear structures such as the nuclear matrix or the nuclear envelope (Varga-Weisz & Becker, 2006). We have identified Brm1 as the likely engine required for the early association of both HP1β and RB with chromatin, and formation of HP1β foci (Fig. 6). We propose that Brm1 activity is necessary for the incorporation of histone variants such as macroH2A into SAHF (Zhang *et al*., 2005). This hypothesis is supported by data indicating that the histone chaperone ASF1a, a critical component in the generation of SAHF, cooperates with Brm1 chromatin-remodeling activity (Moshkin *et al*., 2002).

Intriguingly, ejection of Brm1 from chromatin in senescent cells (Fig. 6A) coincided with its dissociation from HP1β/RB complexes (Fig. 4) and relocalization in complexes of lower molecular weight (Fig. 5). So, why does not Brm1 bind to senescent chromatin? The DNA binding stability of SWI/SNF complexes is stimulated by the presence of hyperacetylated nucleosomes (Hassan *et al*., 2001). Thus, a possible mechanism for Brm1 eviction may be wide-spread hypoacetylation of nucleosomes.

Brm1 cooperates with RB to repress E2F1 and induce complete G_1_ arrest (Trouche *et al*., 1997). Intriguingly, the GST pull-down assays demonstrated that RB does not bind Brm1 in senescent cells (Fig. 4), an event that may be associated with the interference of macroH2A with transcription factor binding and SWI/SNF nucleosome remodeling (Angelov *et al*., 2003). At the end, Brm1 may become dispensable for maintenance of the irreversible growth arrest once nucleosomes have been remodeled.

### The significance of HDAC1 and Brm1 activities for senescence and aging phenotypes

Experimental evidence suggests that both HDAC1 and Rpd3 deacetylases play significant roles in aging and cancer. For example, HDAC1 stimulates angiogenesis in epithelial cells (Kim *et al*., 2001), an event associated with retinal disease and other age-associated pathologies (Gariano & Gardner, 2005), and generates hepatic steatosis in mice (Wang *et al*., 2005), a pathological condition associated with increased age, body weight, and fibrosis (Perumalswami *et al*., 2006).

Brm1 also plays a critical role in tissue aging by forming age-specific repressive complexes containing RB, E2F4, and C/EBPα, a liver transcription factor (Iakova *et al*., 2003). Evidence that Brm1 is crucial for the formation of this repressive complex includes emergence of Brm1/RB/C/EBPα complexes in nuclear proteins from young livers after incubation with excess Brm1, and reduction of Brm1 levels from RB/C/EBPα complexes in old livers after heterochronic parabiosis (Conboy *et al*., 2005).

In sum, our studies provide new mechanistic insights on early events leading to the formation of RB/HDAC1/Brm1 complexes and the onset of senescence in melanocytes and melanocytic nevi. Our results also support the idea that adequate levels of chromatin remodelers are required for maintaining both cell and tissue homeostasis (Bandyopadhyay & Medrano, 2003; Saha & Pahan, 2006), as inhibition or activation of HATs or HDACs can induce senescence in melanocytes (Bandyopadhyay *et al*., 2002; and this communication), apoptosis of melanoma cells (Bandyopadhyay *et al*., 2004) and neurodegeneration (Saha & Pahan, 2006).

## Experimental procedures

### Cell lines and antibodies

Neonatal human melanocytes were isolated and cultured in a manner similar to that described previously (Schwahn *et al*., 2001). The culture medium consists of MCDB-153 medium supplemented with 1 ng mL^−1^ recombinant human basic fibroblast growth factor, 5 µg mL^−1^ insulin, 50 µg mL^−1^ transferrin, 4% fetal bovine serum (Sigma, St. Louis, MO, USA), 6.5 µg mL^−1^ pituitary extract (BioWhittaker, Walkersville, MD, USA), 10 nm cholera toxin, and 0.1 mm 3-isobutyl-1-methylxanthine. The UCD-Mel-N human melanoma cell line (Garcia *et al*., 2004) and the derivative UCD-HDAC1 cells were grown in D/F12 medium [equal amounts of Dulbecco's modified Eagle's medium and F12 medium (Life Technologies, Inc., Rockville, MD, USA)] with 1% antibiotic and antimycotic, 2.5 µg mL^−1^ insulin, 25 µg mL^−1^ transferrin, and 250 ng mL^−1^ epidermal growth factor supplemented with 8% fetal bovine serum.

The following primary antibodies were used: anti-RB antibody (Labvision, Inc, Freemount, CA, USA); anti-HDAC1, antiacetylated histones H3 and H4, antitotal histone H3 and H4 antibodies (Upstate Inc, Lake Placid, NY, USA); anti-Brm1 antibody (BD Transduction laboratories, San Diego, CA, USA); anti-HP1 antibodies (Chemicon, Temecula, CA, USA); and anti-β-actin monoclonal antibody (Sigma), all other antibodies were from Santa Cruz, Inc. (Santa Cruz, CA, USA).

### The Tet-inducible HDAC1 system

*a. Selection of a UCD-Mel-N, Tet-On, G418-resistant cell line*. The UCD-Mel-N cells (Garcia *et al*., 2004) were stably transfected with the rtTA-containing vector using FuGene6 transfection reagent (Roche Applied Sciences, Indianapolis, IN, USA) and 800 µg mL^−1^ neomycin (G418) as the selection marker. Clones were tested for HDAC1 induction levels by transient transfection of the pTRE-Luciferase plasmid (Clontech, Palo Alto, CA, USA). Clones with high inducible luciferase activity were further selected (designated as Tet-on-UCD) for construction of the double transfectant containing the HDAC coding region under the control of the tet-responsive element. *b. Generation of inducible Tet-On cell lines*. A NheI-EcoRV fragment from pOZ-HDAC1 (Humphrey *et al*., 2001) containing the coding region of human HDAC1 was subcloned into the pTRE2-Hyg vector (Clontech). The Tet-on UCD stable clones were selected for resistance to 200 µg mL^−1^ hygromycin. Thirty-five clones were screened for HDAC1 expression. A clone displaying high HDAC1 levels was designated as UCD-HDAC1. Virtually identical results were obtained with two additional clones. Clones expressing empty pTRE-2-hyg vector were designated as UCD-control. Expression of HDAC1, induced by adding 2 µg mL^−1^ of Dox to the culture medium, was followed for 2–3 days or 3 weeks. The culture medium containing Dox was changed three times per week.

#### Cell growth rates

An MTT-assay kit (Roche Applied Science) was used to determine the effect of HDAC1 induction on cell growth. Briefly, 1 × 10^3^ cells of UCD-HDAC1 or UCD-control were seeded on 12-well plates and were treated with 2 µg mL^−1^ of Dox (or left untreated) for increasing time. The MTT assays were performed every other day.

#### Immunofluorescence

The cells grown in glass coverslips were washed twice with phosphate-buffered saline (PBS), fixed in 70 : 30 methanol : acetone mixture for 30 min at –20 °C, and blocked with TBS-T (20 mm Tris, 150 mm NaCl, and 0.2% Tween-20) + 5% nonfat dry milk at 37 °C for 1 h. Incubation with the appropriate polyclonal or monoclonal antibody was performed at 37 °C for 1 h followed by three washes with TBS-T and incubation with an antirabbit secondary antibody conjugated with tetramethylrhodamine isothiocyanate or with an antimouse antibody conjugated with FITC (Santa Cruz) at 37 °C for 1 h. After a final wash, the slides were mounted with antifade mounting media (Vector Laboratories, Burlingame, CA, USA) and visualized under a fluorescence microscope.

### Immunoblotting, immunoprecipitation, and expression of senescent-associated β-galactosidase

Equal amounts (50 µg) of whole cell lysates were electrophoresed in sodium dodecyl sulfate–polyacrylamide gel electrophoresis (SDS-PAGE) and transferred to nitrocellulose membranes. Membranes were washed with TBS-T (TBS + 0.5% Tween-20), blocked with 5% milk in TBS-T for 1 h at room temperature, and incubated overnight at 4 °C in TBS-T containing appropriate dilutions of primary antibodies. The membranes were then washed three times and incubated with a horseradish peroxidase-conjugated donkey antirabbit or sheep antimouse IgG antibody (1 : 3000) in TBS-T for 1 h at room temperature. After washing, the target proteins were detected using an enhanced chemiluminescence immunoblotting detection kit (GE Health Care, Piscataway, NJ, USA). For immunoprecipitation, 600 µg of total cell extracts were incubated with an appropriate antibody at 4 °C for 3 h. Protein G or protein A-Sepharose beads were used to immobilize the antibody complex. The Sepharose-bound immunocomplexes were washed three times with 10 volumes of lysis buffer, boiled in SDS-Laemmli sample buffer, and electrophoresed in SDS-PAGE gels. SA-β-Gal activity was determined as described previously (Dimri *et al*., 1995).

#### Immunohistochemistry

Five-µm thick formalin-fixed paraffin-embedded melanocytic nevi samples were derived from the tissue archives of the Section of Dermatopathology, Department of Pathology, Baylor College of Medicine. Tissue sections were deparaffinized, rehydrated in ethanol, and subject to heat induced antigen microwave retrieval (10 mm citrate buffer pH 6.0; microwaving; 15 min). Deparaffinized sections were incubated with the following antibodies: polyclonal anti-HDAC1 (dilution 1 : 50, Santa Cruz), monoclonal anticyclin A (dilution 1 : 10), monoclonal anti-p16^INK4a^ (dilution 1 : 400), monoclonal anti-Melan A (dilution 1 : 1000) (all from Labvision) in a buffer containing 0.1% bovine serum albumin for 1 h at room temperature. Subsequent immunostaining was performed using the avidin-biotin immunoperoxidase technique following the manufacturer's instruction (Vectastain, Vector Laboratories). Color development with the chromogen 3-amino-9-ethyl carbazole produced a positive red reaction product; hematoxylin was used as a counterstain.

### Histone isolation and fractionation

Histones were isolated by the acid extraction method as described previously (Zweidler, 1978). Isolated histones were fractionated in 15% polyacrylamide gels.

### Expression and purification of GST proteins

GST-tagged RB and its mutant forms were propagated in BL21DE3 (pLysE) cells. Expression and purification of GST proteins were performed using a commercially available GST purification kit (GE Healthcare). For GST pull-down assays, 400 µg of total bacterial lysate containing the GST-tagged protein were incubated with pre-equilibrated glutathione sepharose CL-4B beads at room temperature for 2 h. The immobilized proteins were washed with PBS and incubated with UCD or melanocyte cell extracts for 2 h at 4 ºC. The protein complexes were washed with NP-40 lysis buffer and analyzed in SDS-PAGE followed by immunoblotting.

#### Protein fractionation by size exclusion chromatography

Nuclear extracts were obtained by gentle lysis of cells in buffer A (10 mm Tris–HCl pH 7.5, 150 mm NaCl, 1.5 mm MgCl_2_, and 10 mm EDTA), followed by rupturing the nuclei in high salt-containing buffer B (10 mm Tris–HCl pH 7.5, 420 mm NaCl, 1.5 mm MgCl_2_, 0.5 mm EDTA, and 1 mm DTT). The nuclear extracts were analyzed by size exclusion column SEC-400 (HPLC, Biologic HR; Bio-Rad, Hercules, CA, USA). The individual fractions were separated by SDS-PAGE and detected by immunoblotting. For multiprotein complex identification, the fractions were diluted to adjust the NaCl concentration to 120 mm followed by immunoprecipitation with the indicated antibodies.

#### Isolation of chromatin and analysis of heterochromatin formation

UCD-HDAC1 cells were induced with 2 µg mL^−1^ Dox for 0 day, 3 days, or 3 weeks. The cells on the plate were washed with PBS and overlayed with 2 mL of micrococcal nuclease buffer (250 mm sucrose, 10 mm Tris–HCl pH 7.5, 10 mm NaCl, 3 mm MgCl_2_, 0.1 mm PMSF, and 1 mm CaCl_2_) with 60 U mL^−1^ micrococcal nuclease (Worthington Biochemical Corp., Freehold, NJ, USA) and kept at room temperature for 6 min. The duration of digestion was adjusted to generate a chromatin preparation with a size range of 146 bp–2 kbp. The reaction was stopped by addition of 5 mm EDTA and the cells were promptly harvested. Nuclei were lysed in 10 mm Tris–HCl, 10 mm NaCl, 0.2 mm EDTA, and 0.1 mm PMSF. The resulting suspension was centrifuged at 16 000 ***g*** for 10 min; the supernatant containing the chromatin fraction (oligonucleosomes) was electrophoresed in 1.5% agarose gel.

#### In vitro* binding of HP1β in chromatin*

HP1β was immunopurified from cells using a polyclonal HP1β antibody (Santa Cruz). Sepharose bound HP1β was then mixed with the chromatin fraction using HEPES buffer (10 mm HEPES pH 7.3, 20 mm NaCl, 0.5 mm EGTA, 10% glycerol, and 1 mm DTT) and incubated for 1 h at room temperature. The beads were washed with HEPES buffer followed by proteinase K protein degradation. The HP1β-bound DNA was recovered from beads by phenol : chloroform extraction and alcohol precipitation. The recovered DNA was analyzed in 1.5% agarose gels. *In vivo* HP1β-chromatin binding: the micrococcal digested oligonucleosomal preparation was subjected to DEAE-Sephacel chromatography to exclude the nuclear proteins that are not bound to DNA. After DNA degradation by DNase 1, proteins were analyzed by SDS-PAGE and immunoblotting with an anti-HP1β antibody.

## Supplementary material

The following supplementary material is available for this article:

Fig S1 Irreversibility of the senescent state induced by HDAC1. (A) Induction of HDAC1 protein (see Fig. 1B) results in increased HDAC1 activity. (B) HDAC1 overexpression results in progressive loss of DNA synthesis. (C) Short-term induction of HDAC1 does not cause growth arrest. UCD-HDAC1 cells were seeded in p35mm plastic dishes, left untreated, treated with 2 g μg mL^−1^ Dox to induce HDAC1 for 2 days, or treated with Dox for 2 days followed by removal of the drug for 8 days. SA-β-gal staining (Dimri et al., 1995) was negative in all three conditions. Removal of Dox allowed the cells to proliferate without evident signs of growth alterations or phenotypic changes in their morphology. (D) Long-term induction results in irreversible growth arrest and senescence. Cells treated with Dox for 3 weeks displayed large cells with transparent cytoplasm and were SA-β-gal-positive. Removal of Dox for 8 days did not result in changes of the flat morphology or induce significant proliferation. The cells were fixed with formaldehyde and stained with Crystal violet. (E) The telomerase repeat amplification protocol (TRAP) previously described (Suwa et al., 2001) was used to measure telomerase activity before and after induction of HDAC1 in UCD cells. Two different primary cultures of human normal proliferating melanocytes [NHM (1) and NHM(2)] were used as negative controls. **Experimentals procedures** **HDAC1 Activity Assay.** Cell extracts from UCD-wild-type or UCD-HDAC1 were used to determine RB-associated HDAC activity (HDAC assay kit; Upstate). A SGRGKGGKGLGKGGAKRHRKVLR peptide substrate was prelabeled with [3H]-sodium acetate using benzotriazol-1-yloxytris-(dimethylamino)-phosphonium hexafluoruphosphate. The immunoprecipitates were incubated with the labeled peptide overnight at room temperature. Radioactivity released by the labeled peptide (HDAC activity) was measured after ethyl-acetate extraction. **BrdU labeling.** The BrdU activity assay was performed using a commercially available kit from Roche Applied Sciences. Briefly, UCD-HDAC1 and UCD wild-type cells were seeded on coverslips and treated with or without 2 μg mL^−1^ doxycyclin for the time indicated in the figures. Afterwards, the cells were incubated with 1 : 1000 dilution of BrdU in the fresh medium and the percent of positive cells determined by immunocytochemistry using an anti-BrdU monoclonal antibody provided by the manufacturer. **References** Dimri GP, Lee X, Basile G, Acosta M, Scott G, Roskelley C, Medrano EE, Linskens M, Rubelj I, Pereira-Smith O (1995) A biomarker that identifies senescent human cells in culture and in aging skin *in vivo*. *Proc Natl Acad Sci. USA*, **92**, 9363–9367. Suwa T, Yang L, and Hornsby PJ (2001) Telomerase activity in primary cultures of normal adrenocortical cells. *J Endocrinol.* **170**, 677–684.

Fig S2 Normal human senescent melanocytes display increased HP1β. Left panel: proliferating melanocytes display nucleoplasmic distribution of HP1β. Right panel: HP1β is mostly redistributed in nuclear foci in senescent cells. **Experimentals procedures** Cells were fixed with 10% formaldehyde, washed with PBS containing 0.05% Tween 20 (PBS-T), and permeabilized in 1% Triton X-100 in PBS for 20 min. After incubation with a blocking buffer (5% bovine serum albumin in PBS), the cells were exposed to anti-HP1β antibody (monoclonal antibody 3448, CHEMICON) followed by incubation with Alexa Fluor Alexa Fluor 488 (Invitrogen; Molecular Probes, Eugene, OR, USA) secondary antibodies. After a final wash, the slides were mounted with antifade mounting media containing DAPI (H-1200, Vector Laboratories) and observed with an inverted fluorescence microscope. This material is available as part of the online article from: http://www.blackwell-synergy.com/doi/abs/10.1111/j.1474-9726.2007.00308.x (This link will take you to the article abstract). Please note: Blackwell Publishing are not responsible for the content or functionality of any supplementary materials supplied by the authors. Any queries (other than missing material) should be directed to the corresponding author for the article.
